# The Regulation of Steroid Action by Sulfation and Desulfation

**DOI:** 10.1210/er.2015-1036

**Published:** 2015-07-27

**Authors:** Jonathan W. Mueller, Lorna C. Gilligan, Jan Idkowiak, Wiebke Arlt, Paul A. Foster

**Affiliations:** Centre for Endocrinology, Diabetes, and Metabolism, Institute of Metabolism and Systems Research, University of Birmingham, Birmingham B15 2TT, United Kingdom

## Abstract

Steroid sulfation and desulfation are fundamental pathways vital for a functional vertebrate endocrine system. After biosynthesis, hydrophobic steroids are sulfated to expedite circulatory transit. Target cells express transmembrane organic anion-transporting polypeptides that facilitate cellular uptake of sulfated steroids. Once intracellular, sulfatases hydrolyze these steroid sulfate esters to their unconjugated, and usually active, forms. Because most steroids can be sulfated, including cholesterol, pregnenolone, dehydroepiandrosterone, and estrone, understanding the function, tissue distribution, and regulation of sulfation and desulfation processes provides significant insights into normal endocrine function. Not surprisingly, dysregulation of these pathways is associated with numerous pathologies, including steroid-dependent cancers, polycystic ovary syndrome, and X-linked ichthyosis. Here we provide a comprehensive examination of our current knowledge of endocrine-related sulfation and desulfation pathways. We describe the interplay between sulfatases and sulfotransferases, showing how their expression and regulation influences steroid action. Furthermore, we address the role that organic anion-transporting polypeptides play in regulating intracellular steroid concentrations and how their expression patterns influence many pathologies, especially cancer. Finally, the recent advances in pharmacologically targeting steroidogenic pathways will be examined.

- Introduction
  - Steroid analysis
- Steroid Sulfatases
  - Molecular overview and functionality
  - STS cellular and tissue distribution
  - The regulation of STS
- Steroid Sulfotransferases and PAPS Synthases
  - Molecular overview and functionality
  - Tissue and cellular distribution
  - Regulation of sulfotransferases and PAPS synthase activity
- Cellular Influx and Efflux of Sulfated Steroids
  - OATP-regulated influx
  - MRP-regulated efflux
  - Estrone sulfate influx and efflux
  - DHEAS influx and efflux
  - Genetic variation and regulation of OATP expression
- Disease-Causing Mutations Affecting Steroid Sulfation and Desulfation
  - Pathogenic mutations in steroid sulfatases and SUMF1
  - Pathogenic mutations in steroid sulfotransferases and PAPS synthases
- Dysregulation of Steroid Sulfation and Desulfation
  - Cancer
  - Aging
- Pharmacological Intervention
  - STS inhibitors
  - Modulation of sulfation
- Future Directions

## I. Introduction

**Figure 1.**
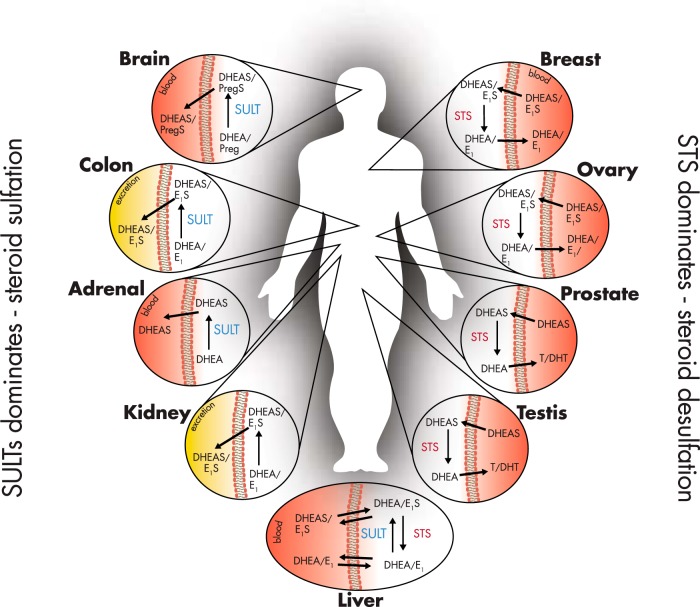
Predominance for steroid sulfation or desulfation in endocrine and selected nonendocrine human tissues. Sulfation pathways dominate in the healthy brain, colon, adrenal, and kidney. The colon and kidney sulfate steroids to expedite excretion. The adrenal synthesizes DHEA, which is subsequently sulfated to increase water solubility and allow circulatory transport. The brain favors sulfation, although this is primarily due to the role of pregnenolone sulfate as a neurosteroid. In the liver, a so-called “futile-loop” of DHEA/DHEAS, E_1_/E_1_S, and E_2_/E_2_S occurs, as well as other steroids. Because sulfated forms of these steroids persist longer in the circulation due to greater half-lives, this accounts for their higher circulating concentrations compared to their nonsulfated forms. Desulfation, via STS, dominates in the breast, ovary, prostate, testis, placenta (not shown), and uteri (not shown). In breast and ovarian tissue, E_1_S uptake occurs through OATPs (see *Section IV*), where it is desulfated by STS to form E_1_, and subsequently E_2_ via 17βHSDs. In the prostate and testis, circulating DHEAS can also be transported into the cell via OATPs, desulfated by STS, and then metabolized to androgens such as T and DHT, which can then enter the circulation.

Sulfation and desulfation are vital biological processes that regulate steroidogenesis and thus, steroid hormone action in a variety of tissue (Figure 1). Controlled by two distinct enzyme families, the sulfatases and the sulfotransferases (SULTs), these processes are intimately involved in the hydrolysis and esterification of sulfate groups to alkyl (eg, dehydroepiandrosterone [DHEA]) and aryl (eg, estrone [E_1_]) steroids. As early as the 1940s, steroids were identified as one of the major classes of biomolecules that could be sulfated (1–3). Chemically, it is possible to attach a sulfate to each and every hydroxyl group of a steroid, and taking into account the astonishing substrate promiscuity of the various sulfotransferase enzymes, many different sulfated steroids are detected analytically in biological samples (4). Historically, sulfated steroids were considered to be metabolic end products because their increased water solubility expedites excretion. However, over the past 20 years, a wealth of research demonstrates that sulfated steroids, such as DHEA sulfate (DHEAS) and E_1_ sulfate (E_1_S), can act as circulating reservoirs for the peripheral formation of bioactive hormones. Therefore, an understanding of how sulfation and desulfation processes are regulated and dysregulated provides key insights into physiological and pathophysiological endocrine control. This review examines our current understanding of sulfation and desulfation steroid pathways, including the intracellular influx and efflux of sulfated steroids via the organic anion transporter proteins (see *Section IV*), the role of these pathways in disease (see *Sections V and VI*), and the potential to pharmacologically target these pathways for therapeutic gain (see *Section VII*).

### A. Steroid analysis

The era of steroid analysis via immunoassay is drawing to a close as these unspecific assays are replaced by high-throughput, specific, sensitive mass spectrometry (MS) analyses (5). The inherent problem of immunoassays is their poor specificity due to antibody cross-reactivity, which hampers both enzyme immunoassay and RIA approaches. With regard to the measurement of estradiol (E_2_), this problem was identified over 25 years ago (6) and more recently in human plasma samples (7). However, with the increasing clinical and laboratory demand for steroid measurements, cheap RIA kits emerged as popular one-step kits and multiplex assays in the 1980s and 1990s. These “direct” immunoassay kits sacrificed accuracy for speed and economy (8).

Gas chromatography (GC)-MS, coupled with either electron impact ionization or chemical ionization, is sensitive and specific, but it requires extensive sample cleanup as well as multistep deconjugation and derivatization procedures. Thus, it is liquid chromatography (LC)-MS or LC-tandem MS (LC-MS/MS) that after pioneering work in the 1990s (9) is becoming the reference method for the analysis of both sulfated and nonsulfated steroids in clinical laboratories, due to its fast turnaround time and high accuracy. Indeed, a recent statement by The Endocrine Society had attempted to implement a policy toward introducing LC-MS as the diagnostic standard for publication of steroid measurements (8), although this position was later relaxed because many laboratories do not have the technology to achieve such accurate analysis (10). Table 1 shows plasma reference ranges for nonsulfated and sulfated steroids in adult men, premenopausal adult women, and postmenopausal women.

**Table 1.** Approximate Estimates of Plasma Concentrations of Steroids and Their Sulfates in Human Adults

Steroid	Males	Premenopausal Females	Postmenopausal Females
Cholesterol	0–1 nmol/L	0–1 nmol/L	0–1 nmol/L
Cholesterol sulfate	0–3 μmol/L	0–3 μmol/L	0–3 nmol/L
Pregnenolone	1–15 nmol/L	1–15 nmol/L	1–15 nmol/L
Pregnenolone sulfate	200–1000 nmol/L	100–1000 nmol/L	10–500 nmol/L
DHEA	10–25 nmol/L	5–30 nmol/L	2–20 nmol/L
DHEAS	2–10 μmol/L	1–8 μmol/L	1–6 μmol/L
Androsterone	2–4 nmol/L	2–4 nmol/L	
Androsterone sulfate	0–5 μmol/L	0–1 μmol/L	
E_1_	30–140 pmol/L	15–500 pmol/L	10–120 pmol/L
E_1_S	2–4 nmol/L	2–5 nmol/L	0.5–2 pmol/L
E_2_	20–40 pmol/L	5–1000 pmol/L	5–80 pmol/L
Progesterone	0–0.4 nmol/L	0–80 nmol/L	0–0.4 nmol/L
T	5–25 nmol/L	0.2–2 nmol/L	0.2–1 nmol/L
DHT	850–3500 pmol/L	80–1300 pmol/L	30–650 pmol/L
DHTS	50–100 nmol/L		

Where values are missing, not enough sufficient evidence is available to provide accurate estimates.

The measurement of sulfated steroids can be straightforward, as conjugated steroids easily ionize resulting in greater LC-MS sensitivity. RIAs do exist that can measure sulfated steroids, as mentioned above cross-reactivity and the lability of the sulfate group, make these methods unreliable. Advances employing ultrahigh pressure LC quadrapole time-of-flight MS can now detect a range of sulfated and glucuronidated steroids simultaneously in human urine with similar sensitivity to GC-MS (11). With regard to plasma, recently a rapid LC-MS/MS procedure has been designed involving diethylether extraction from plasma, purification by immunosorbents containing specific antibodies against E_1_S, followed by LC-MS/MS using electrospray ionization. This sample preparation markedly improved the sensitivity of LC-MS/MS for E_1_S (12). Others have utilized LC-MS/MS with electrospray ionization to detect other sulfated steroids such as dihydrotestosterone sulfate (DHTS) and 3β-hydroxy-5α-androstane-17β-sulfate simultaneously (13). However, the main difficulty with measuring most sulfated steroids lies with the lack of availability of appropriate reference standards, making measurements impossible to accurately quantify.

## II. Steroid Sulfatases

### A. Molecular overview and functionality

The sulfatase enzyme family catalyzes the hydrolysis of sulfate ester bonds from a wide range of substrates. Within this family, 17 genes have been identified in humans, many associated with genetic disorders (14). Of these, three have their crystal structure determined: arylsulfatase A, B, and C (the latter also known as steroid sulfatase [STS]). Arylsulfatases A and B are both water soluble and involved in the hydrolysis of cerebroside-3-sulfate and the breakdown of glycosaminoglycans (GAGs), respectively; thus, neither is involved in steroid pathways. In contrast, STS has been shown to be the primary enzyme involved in steroid desulfation (15) and therefore is the main focus in this review.

The principal hormone substrates for STS are E_1_S, DHEAS, pregnenolone sulfate, and cholesterol sulfate, and therefore this enzyme represents one of the major pathways in regenerating biologically active steroids in both steroidogenic and nonsteroidogenic tissues. DHEA and E_1_ circulate predominantly in their inactive sulfated forms, DHEAS and E_1_S, respectively. Cells can transport, via organic anion-transporting polypeptides (OATPs; see *Section IV*), circulating hydrophilic sulfated steroids, such as DHEAS and E_1_S, for intracellular desulfation by STS and subsequent generation of androgenic and estrogenic steroids.

Structurally, STS has a hydrophobic domain and is a membrane-bound microsomal enzyme, mainly localized in the rough endoplasmic reticulum (16, 17). The 10 exons- spanning STS gene is located on the short arm of chromosome X and mapped in Xp22.3-Xpter (17–19). It escapes X-inactivation (20) with a nonexpressed Y-linked homolog in man (18). It is thought that STS is glycosylated, with its three-dimensional structure crystallography showing it to be a monomer of a “mushroom-like” shape with two hydrophobic antiparallel α-helices protruding from a spherical molecule (21, 22). This 40 Å-long hydrophobic stem is most likely embedded in the luminal membrane of the endoplasmic reticulum. Opening beside it is a long narrow pocket with the enzyme reaction site lying at the base, suggesting that the product has to travel through the endoplasmic reticulum membrane (23).

STS is expressed as a membrane-associated precursor with a molecular mass of 63 kDa and asparagine-linked oligosaccharide chains. These chains are cleaved by endoglucosaminidase H, creating a final size of 61 kDa with a half-life of 4 days (24). STS can undergo various post-translational modifications; it holds four potential N-glycosylation sites; however, digestion by endoglycosidase H and endoglucosaminidase H showed that only two (Asn47 and Asn259) are used (25, 26). Supporting this, Stengel et al (27) found that although all four of the N-linked sites are glycosylated to some extent, only mutations in two major glycosylation sites, again at asparagines 47 and 259, decreased activity. Another modification is the conversion of C75 to formylglycine (FGly) (see *Section II.A,1*) and further hydration forms the gem-diol hydroxylformylglycine with a bound sulfate in the resting state (28).

Disease resulting from impaired STS activity, such as X-linked ichthyosis (XLI), is most often due to large deletions of the gene (80–90%). Alternatively, in some XLI patients, six-point mutations have been identified, all abolishing STS activity (29–31). Five of the point mutations lead to nonconservative amino acid changes, and the sixth is a frameshift mutation. Interestingly, these mutations are all within 105 residues of each other in the C-terminal half. Two are even on the same amino acid, 372, changing tryptophan to either arginine or proline. The others are an arginine for tryptophan at amino acid 444, a tryptophan for a cysteine at 446, a cysteine substitute for a leucine at 341, and an arginine for serine at 419. This close accumulation of mutations suggests that this as an area crucial for STS activity (32, 33). Furthermore, artificially truncating N or C termini of the STS enzyme does not have any effect on protein synthesis and degradation, when transfected into COS-1 cells, however, there was reduction in activity (34). Thus, when coexpressed with wild-type STS, C-terminal STS mutants have a dominant negative effect.

#### 1. Sulfatase-modifying factors

**Figure 2.**
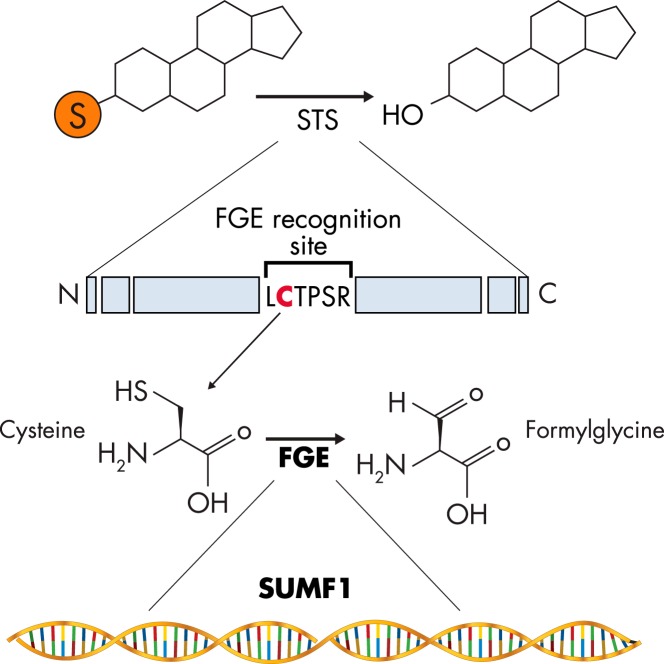
SUMF1 and FGE. SUMF1 encodes for the enzyme FGE, which catalyzes the conversion of cysteine to FGly found at the FGE-recognition site LCTPSR on STS. This reaction results in increased steroid desulfation by elevated STS activity.

The molecular mechanisms underlying STS catalytic activity are highly conserved among different human sulfatase enzymes (16, 35). A cysteine residue resides in the catalytic center of all sulfatases, which is post-translationally modified to form a FGly residue (Figure 2). FGly is catalytically active and “attacks” the sulfate moiety of substrates; it is essential to bind the substrate and also to hydrolyze the sulfate ester bond (36, 37).

Modification of the cysteine to form FGly is mediated by the coenzyme FGly-generating enzyme (FGE), which is encoded by the sulfatase-modifying factor 1 (*SUMF1*) gene. FGE, a glycosylated enzyme that, like STS, resides in the endoplasmic reticulum, can be secreted by cells (38). Intriguingly, FGE can thus act in a paracrine fashion because it can be taken up by neighboring cells as a functional protein and increase intracellular sulfatase activity (39). The importance of this process in regulating STS activity and steroid output is currently unknown.

Mutations in *SUMF1* cause multiple sulfatase deficiency, a rare and fatal autosomal recessive disorder characterized by absent activity of all sulfatase enzymes (see *Section V.A*) (40, 41). A paralog of *SUMF1*, *SUMF2*, has been cloned in vertebrates due to its sequence homology to *SUMF1* (42, 43). SUMF2 lacks the crucial catalytic domain present and highly conserved in *SUMF1*, and the role of *SUMF2* in the process of post-translational modification of sulfatases is, at present, unresolved.

### B. STS cellular and tissue distribution

STS is a membrane-bound protein primarily localized in the lumen of the endoplasmic reticulum (23), although it has also been found in Golgi cisternal, trans-Golgi reticulum, plasma membranes, and elements of the endocytic pathway (44). In 1965, Warren and French (45) examined STS tissue distribution and found virtually ubiquitous expression in human tissues, with placenta demonstrating the greatest mRNA and activity. These findings have been substantiated by many research groups using various techniques, such as immunohistochemistry, biochemical analysis, and real-time PCR, analyzing a multitude of tissues including testis, ovary, adrenals, prostate, skin, brain, endometrium, kidney, thyroid, pancreas, colon, aorta, bone, and lymphocytes (19, 35, 46), which all show STS activity.

From gestation and throughout life, STS activity remains imperative to both genders for tissue-specific steroid hormone production and regulation. In premenopausal women, the main source of active E_2_ is the ovaries, whereas E_1_ is formed mostly in peripheral tissues, eg, fat. However, in postmenopausal women and men, E_2_ is metabolized from adrenal steroid precursors at extragonadal sites such as breast and fat. The active estrogens can be generated by two enzymes, aromatase and sulfatase. STS desulfates E_1_S to E_1_, followed by reduction to E_2_ via reductive 17β-hydroxysteroid dehydrogenase (17βHSD) activity. Aromatase converts androstenedione and T to E_1_ and E_2_, respectively. Of note, androstenedione is synthesized from the precursors DHEA and DHEAS, which circulates at very high concentrations compared to other steroids (see Table 1). STS desulfates DHEAS, and thus STS also plays a role in liberating androgens for aromatization (47).

### C. The regulation of STS

STS tissue activity fluctuates depending on physiological conditions, but exactly which factors regulate these changes remains unknown. For example, STS activity is higher in leukocytes in the third trimester of human pregnancy compared to nonpregnant females and adult males (48), an effect possibly regulated by elevated FSH concentrations (49). Furthermore, and again as measured in leukocytes, STS activity changes throughout puberty, differing between males and females and being at its highest in prepubertal females (50). STS is also frequently increased in various malignant tissues, such as in breast cancer (see *Section VI.A.1*). However, very little is known about the underlying regulation of this expression or activity, although circulating estrogen concentration most likely plays a role.

The promoter region and 5′ upstream regulatory elements of the *STS* gene were first characterized in human placenta (51); however, this promoter was noted to lack basal activity, suggesting additional regulatory elements. Subsequently, tissue-specific STS isozymes with different kinetic parameters for DHEAS and E_1_S were discovered (52–54). Zaichuk et al (52) characterized the 5′ heterogeneity of the human STS gene in MCF7 cells. The *STS* gene exhibits alternative splicing and promoter usage, which is likely to be the basis for tissue-specific regulation. 5′-Rapid amplification of cDNA ends analysis has identified eight splice variants used in *STS* transcription based on the first six exons. First reported was exon 1a from placenta, which utilizes DHEAS as the major steroid produced by fetal adrenal glands and the main source of active estrogens (55). All splice variants encode the same active protein and all, except exon 1d which is found only in peripheral mononuclear leukocytes, vary in length with multiple transcription start sites with tissues generally expressing one or more of these variants. Heterogeneity in signal peptide sequences is thought to facilitate folding and localization of proteins to the correct intracellular compartment (19, 46).

STS mRNA and activity are higher in many cancerous tissues compared to normal, implying an important role in hormone-dependent tumor growth (see *Section VI*). Although ubiquitously expressed, the regulation of STS expression does appear to be tissue specific and is subjected to various feedback mechanisms, such as that shown by the positive correlation between STS and estrogen receptor (ER) isotypes mRNA (52). In MCF7 cells, *STS* transcription may be up-regulated by E_2_ via direct binding to ER and activation of estrogen response elements in the *STS* promoter regions. Furthermore, MCF7 cells treated with antiestrogen ICI182780 displayed reduced basal and E_2_-stimulated expression of all STS mRNA. E_2_ also induced ERα degradation in an autoregulatory feedback loop, whereas pretreatment with proteasomal inhibitor MG132 prevented this. Exposure to E_2_ and MG132 resulted in STS mRNA increase, whereas MG132 alone reduced STS mRNA (52, 56). Thus, to control estrogenic tissue, *STS* expression may be regulated by local estrogen concentrations in an ER-dependent manner. However, as yet, this pathway for STS regulation has not been demonstrated in other cell lines, suggesting that it may be unique to MCF7 cells.

In addition to the potential for estrogens to regulate STS activity, the proinflammatory cytokines IL-6 and TNFα alter STS enzyme kinetics. MCF7 cells increase STS activity in response to IL-6 and TNFα without alteration in STS mRNA levels (57, 58), a trait also noted in other cancer cell lines (59). This suggests that post-translational modifications, possibly via STS glycosylation, are involved in regulating STS activity (17, 60, 61). However, it cannot be currently ruled out that these cytokines alter membrane permeability and therefore increase substrate availability, which is then perceived as an increase in STS activity (62).

Regulation of STS by inflammatory mediators is of interest, considering that sex steroids have a role in immune functions, inflammatory processes (63, 64), and cancer, where STS activity is frequently dysregulated and often associated with inflammation (65). Both epidemiological and immunological evidence implies that steroids can influence the pathogenesis of many chronic inflammatory diseases (66). For example, in the vascular smooth muscle cells of atherosclerosis patients, STS was found to be higher in females with mild atherosclerotic changes compared to severe disease and male aortas. Additionally, the counterpart of STS, estrogen sulfotransferase (SULT1E1), was lower in females with severe disease (67), suggesting the importance of the STS/SULT ratio in the local regulation of estrogen formation in inflammatory disease states. How this alteration in ratio affects disease inflammatory progression remains ill-defined.

## III. Steroid Sulfotransferases and PAPS Synthases

### A. Molecular overview and functionality

Endocrine sulfation pathways include sulfate uptake, conversion of this inert anion to active sulfate in the form of 3′-phospho-adenosine-5′-phosphosulfate (PAPS), and transfer to steroid hydroxyl groups by sulfotransferases. Sulfate is an obligate nutrient provided mainly by food and drinking water, taken up from the gut by several sulfate transporters of the solute-linked carrier (SLC) 13 and 26 gene families (68), and to a minor extent also by oxidation of cysteine and methionine amino acids (69).

**Figure 3.**
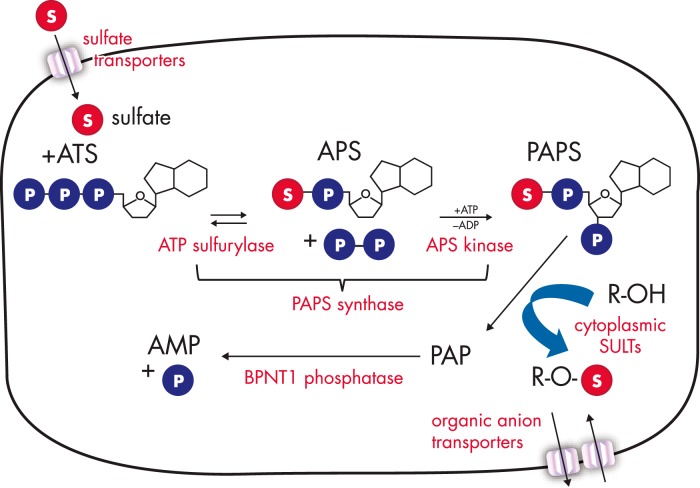
Human sulfation pathways are complex. The various parts of human sulfation pathways are schematically depicted. Several sulfate transporters are responsible for cellular sulfate uptake (reviewed in Refs. 59 and 62), followed by the two-step enzymatic sulfate activation by bifunctional PAPS synthases. PAPS is then either used directly by cytoplasmic and nuclear sulfotransferases or shuttled to the Golgi apparatus to serve a multitude of Golgi-residing carbohydrate and protein sulfotransferases. In contrast to the nonsulfated biomolecules, sulfated xenobiotics or steroids need designated organic anion transporters to enter or exit cells. Many different sulfatases exist to cleave sulfate esters again. The otherwise toxic, sulfation by-product PAP needs to be removed by dedicated phosphatases (reviewed in Ref. 65). In this review, we focus on sulfate activation, steroid sulfation, and desulfation as well as the transport of steroid sulfates via organic anion transporters. For all other steps, the reader may refer to the reviews given above.

Enzymatic sulfate activation by PAPS synthase is essential due to the inert nature of the sulfate ion; this activation occurs via consecutive enzymatic steps (Figure 3) (70, 71). First, the AMP moiety of ATP is transferred to sulfate catalyzed by the ATP sulfurylase activity of PAPS synthase, yielding adenosine-5′-phosphosulfate (APS). Formation of this unusual phospho-sulfo-bond is highly endergonic, so that subsequent cleavage of the release pyrophosphate by ubiquitous pyrophosphatases and an additional phosphorylation step are needed to draw the reaction to completion. This phosphorylation of APS at its ribose 3′-hydroxyl group is carried out by the APS kinase domain of PAPS synthase, resulting in 3′-phospho-APS (PAPS) (70). PAPS is the universal sulfate donor required by all human sulfotransferases, and in humans and most vertebrates it is exclusively produced by two bifunctional PAPS synthases, PAPSS1 and PAPSS2 (72). Active sulfate in the form of PAPS is used by sulfotransferases for sulfation of a multitude of hydroxyl and amino groups in a diverse array of biomolecules, including steroids. The by-product of this reaction, the bis-phospho-nucleotide 3′-phospho-adenosine-5′-phosphate (PAP), is then degraded by dedicated phosphatases (73, 74) (see *Section III. C.*).

Sulfotransferases are a large gene family traditionally classified into membrane-bound, Golgi-residing enzymes (75) and soluble, cytoplasmic sulfotransferases (76). Golgi-residing sulfotransferases are responsible for sulfation of proteins, carbohydrates, and proteoglycans, whereas cytoplasmic sulfotransferases modify mainly hydrophobic, low-molecular-weight substances such as phenols, xenobiotics, and steroids. Recent research has provided an increasing number of structural studies on cytosolic sulfotransferases, but also with Golgi sulfotransferases, eg, the carbohydrate 2-O (77) and 3-O-sulfotransferase (78) as well as the first structure of a protein sulfotransferase—the human TPST2 protein (79). Sequence conservation is rather low between these different sulfotransferases, but their fold and catalytic features including binding of the PAPS cofactor are highly conserved. Central to all sulfotransferases is an α/β-motif consisting of a five-stranded parallel β-sheet; the 5′ phosphosulfate loop-loop consisting of a strand-loop-helix structure, which is involved in binding the phosphosulfate moiety of the PAPS cofactor; and an additional conserved α-helix (80). Ensembl lists 62 sulfotransferase genes within the human genome (including four pseudogenes) (81) (Ensembl release 76). Sixteen of these represent cytoplasmic sulfotransferases, and five of these are associated with steroid sulfation: SULT1A1, SULT1E1, SULT2A1, as well as the two isoforms of the *SULT2B1* gene, SULT2B1a and SULT2B1b (Table 2) (82).

**Table 2.** Sulfotransferases and Their Steroid Substrate

Steroid	SULT	K_m_ Values	Refs.
DHEA	SULT2A1	0.8–3.7 μm	102, 120, 313, 414–418
	SULT1E1	0.2 μm	419
Androsterone	SULT2A1	2.1 μm	415
Pregnenolone	SULT2A1	1.9−4.9 μm	102, 313
	SULT2B1a	4.4 μm	102
E_1_	SULT1E1	0.2 μm	419
E_2_	SULT1E1	4–300 nm	118, 121, 418–420
	SULT1A1	240 nm	421
Cholesterol	SULT2B1b	1.2 μm	102

Physiological studies on SULTs are hampered because sulfotransferase repertoires are different between mouse and man; thus, findings in mice cannot always directly be translated to human physiology. Only 46 of the above-mentioned 62 human sulfotransferases have a direct counterpart in mice. Although SULT3 genes are expressed in rodents, there is merely a nonfunctional SULT3 pseudogene in humans. Within mammals, a SULT5A1 gene can be found in rodents, but has been lost from all other mammalian genomes (81) [Ensembl release 76; ENSMUSG00000000739]. Furthermore, a single copy of the SULT2A1 gene in humans contrasts with a large gene cluster in mice (SULT2A1-SULT2A7), possibly explaining the absence of a suitable SULT2A1 knockout model. On the other hand, whereas there is only one SULT1A gene in mice, the SULT1A gene family forms a genomic cluster in humans at chromosome 16p11.2, with one gene duplication into 1A1 and 1A3 type proteins within simians about 42 million years ago and two further gene duplications in hominines (about 8 million years ago) resulting in the four 1A genes found in chimpanzees and humans (81) [Ensembl release 76; ENSG00000196502]. SULT1A3 and SULT1A4 encode identical proteins, and a unique glutamate residue at position 146 drives these sulfotransferases toward sulfation of catecholamines (83). Interestingly, specification at the human 16p11.2 locus does not stop here because for the SULT1A1 gene, interindividual differences in gene copy number have been described, with some individuals carrying up to five SULT1A1 gene copies correlating with elevated SULT1A1 activity (84).

Cytosolic SULTs generally show broad substrate specificity. Taking the metabolic capacity of the microbiota additionally into account (85), virtually unlimited numbers of substrates may be sulfated. Traditionally, certain sulfotransferases were named according to their presumably preferred substrate, eg, estrogen sulfotransferase (SULT1E1) and DHEA sulfotransferase (SULT2A1). In light of the greatly overlapping affinities of different steroids to different SULTs (Ref. 86 and Table 2), the most likely sulfotransferase for E_2_ sulfation may still be SULT1E1 (because SULT1A1 and SULT1A3 have much lower affinities for estrogens, with maximal activity in the micromolar range). DHEA, however, may also be sulfated by SULT1E1 or SULT2Bs, in addition to SULT2A1. On the other hand, SULT2A1 sulfates several other steroids as well as many xenobiotics. A comprehensive study compared ligand-binding profiles for eight human SULTs (87); out of SULT1C-1 to -3, SULT1B1, SULT1A1, SULT1A3, SULT2A1, and SULT1E1, E_1_ only bound to SULT1E1; 2-hydroxyestradiol only bound to SULT1C3, 4A1, 2A1, and 1E1; DHEAS only bound to SULT2A1 and 1E1; and the bile acid lithocholic acid only bound to SULT2A1 and 1E1.

The broad substrate specificity of the sulfotransferase enzymes may be linked to three highly flexible loops flanking the catalytic binding site that can adapt to various ligands. These loops are the least conserved parts between different sulfotransferases. One of them, Asn226-Gln244 in SULT2A1, is referred to as a “cap that closes in,” once the PAPS cofactor is bound with Arg247 (conserved in all SULTs) making direct contact to this nucleotide (88). This gating mechanism confers substrate specificity (89), and the equilibrium between open and closed conformations may restrict access to the catalytic core for larger ligands, whereas sulfation of smaller substrates is unaffected (88). Active site plasticity may be a general feature of SULT enzymes (90), and it has two direct consequences for the interaction of SULT2A1 with steroid molecules. First, the steroid molecule may bind in a nonproductive way causing substrate inhibition (91). Second, for some pseudosymmetric steroids with two hydroxyl groups, the substrate plasticity of SULTs allows sulfation also at other hydroxyl groups than the normally targeted 3-hydroxyl group of the steroid A-ring. Interestingly, this change in stereoselectivity may happen in SULT2A1 upon allosteric binding of certain drugs, eg, celecoxib, a cyclooxygenase-2 inhibitor (92). Furthermore, bis-sulfated steroids may be created in this way that represent poorer substrates for STS (93). Given this substrate promiscuity of sulfotransferases, it is essential to understand the regulation of tissue-specific expression of the different *SULT* genes.

### B. Tissue and cellular distribution

Sulfotransferase enzymes are broadly expressed in the human body. Tissues that putatively have the highest sulfation activities are those that are affected most severely by loss of the ubiquitously expressed 3′,5′-bisphosphate nucleotidase (BPNT1) phosphatase, the enzyme that removes cytoplasmic PAP, the otherwise toxic by-product of sulfation, by degrading it into AMP and phosphate. In the BPNT1 knockout mouse model, the tissues mainly affected are hepatocytes as well as enterocytes of the early small intestine and proximal tubule epithelial cells of the kidney (94); however, it should be noted that adrenal steroid synthesis in these knockout animals was not investigated.

The expression of five sulfotransferases (SULT1A1, SULT1A3, SULT1B1, SULT1E1, and SULT2A1) was recently compared in four human tissues (liver, intestine, kidney, and lung) by quantitative Western blotting (95). The highest concentrations of sulfotransferases were found in liver and intestine consistent with the above, with SULT1A1/SULT2A1 and SULT1B1/SULT1A3+A1 the most/second most prevailing SULTs in these tissues (95). SULT1E1 has been identified as the major sulfotransferase in lung tissue, whereas expression is at lower levels in liver and intestine and nonexistent in the kidney (95). SULT1E1 may play a more important role during fetal development, being highly expressed in fetal liver and lung (96, 97). SULT1A1 and SULT1B1 were found in all four tissues tested; SULT1A3 was found in kidney, lung, and intestine, but not in liver (95). Therefore, SULT2A1 may exclusively carry out hepatic sulfation of orally administered and externally absorbed DHEA.

Within the human adrenal cortex, SULT2A1 is specifically expressed in the zona reticularis (98, 99), and hence this sulfotransferase is responsible for the massive DHEAS production in this tissue. Strong adrenal expression of SULT2A1, compared to SULT2B1a and SULT2B1b, was also reported by Javitt et al (100). Thus, one may regard SULT2A1 as a gene with dual functionality, detoxification of xenobiotics in the liver and maintaining steroid homeostasis in the adrenal; its secondary adrenal function may have been gained only during primate evolution (101).

All of these sulfotransferases need to be provided with active sulfate in the form of PAPS, and hence the coexpression of at least one of the two PAPS synthase genes is crucial for their functionality. The *PAPSS1* gene is thought to be expressed ubiquitously (82, 102), whereas *PAPSS2* seems to be expressed in a tissue-specific manner, with particularly high expression in the adrenal glands, colon, lung, and liver. *PAPSS2* gene expression seems to be more dynamically regulated (103–105).

### C. Regulation of sulfotransferases and PAPS synthase activity

Sulfotransferase genes are part of the phase-II-biotransformation machinery targeting drugs and xenobiotics, and as such their transcriptional regulation (mainly of SULT1A1 and SULT2A1) is highly complex, involving regulation by several nuclear receptors like the pregnane X receptor (PXR) and the constitutive androstane receptor (CAR) (106). These receptors are activated by xeno- and endobiotics, and they also regulate the expression of many other detoxification genes like cytochromes P450 and uridine 5′-diphospho-glucuronosyltransferases (107). What makes sulfotransferases special in this regard is that the ligands activating those nuclear receptors are substrates for sulfation, and this sulfation usually decreases ligand binding to the respective nuclear receptor, representing a crucial feedback regulation loop. Noteworthy, sulfation may convert some nuclear receptor ligands into effective receptor antagonists. This phenomenon, well described for oxysterols and their involvement in the regulation of bile acid detoxification and ultimately lipid metabolism, is further described in *Section V.B.2*.

The transcriptional regulation of *SULT* gene expression by nuclear receptors may even result in cross-talk between different steroid hormones. In this regard, induction of the cholesterol-preferring sulfotransferase SULT2B1b by the vitamin D receptor was recently shown (108). Furthermore, glucocorticoids may antagonize estrogen function by glucocorticoid receptor-mediated transcriptional up-regulation of estrogen sulfotransferase SULT1E1 (109, 110), resulting in inactivating sulfation of E_2_.

Many studies on transcriptional regulation of SULTs have focused on the *SULT2A1* gene (111, 112). In fact, in a mouse model for hyposulfatemia due to disruption of the NaS1 sodium sulfate cotransporter, SULT2A1 is the only sulfotransferase that shows significant changes in expression (113). Interestingly, transcriptional coregulation of the genes for SULT2A1 and the producer of active sulfate, PAPSS2, has been shown in some cases (103, 104). The murine *Sult2a1* gene may also be coregulated with the DHEAS efflux transporter *Mrp4* through the nuclear receptor CAR, with *Mrp4* knockdown reducing *Sult2a1* expression and CAR activation increasing both *Sult2a1* and *Mrp4* (114).

Most studies on xenobiotic-induced transcriptional up-regulation of SULTs focus on hepatic detoxification pathways, mainly in rodent models. In human adrenal cells, *SULT2A1* gene expression is increased upon stimulation by CRH or ACTH (115) and regulated by the nuclear receptor steroidogenic factor 1, the transcription factor GATA-6 (116), and ERα (98). Although binding of all these transcription factors to the human SULT2A1 promoter has clearly been demonstrated, this still does not explain the striking specificity of SULT2A1 expression within the human zona reticularis or the remarkable changes in SULT2A1 expression directly after birth, during adrenarche, and in human aging.

On the protein level, SULTs are subject to substrate inhibition (eg, DHEA binding to SULT2A1). SULTs are usually exposed to different substrates at the same time. Some xenobiotics are able to bind to the mostly hydrophobic ligand binding sites of SULTs, thereby blocking enzyme activity. This mechanism may explain the hormone-like, estrogenic action of endocrine disruptors that otherwise do not bind and activate the ER (117). Estrogen action can be enhanced by the potent inhibition of SULT1E1, resulting in reduced estrogen inactivation by sulfation, mediated by hydroxylated metabolites of polyhalogenated aromatic hydrocarbons (118). As an example, tetrabromobisphenol A, a commonly used flame retardant, mimics E_2_ binding to SULT1E1, making use of the versatile substrate binding pocket and inhibiting the activity of the enzyme (119). These findings highlight the potential of xenobiotics to cause endocrine disruption by interfering with steroid sulfation without the need to bind to hormone receptors directly.

It is well established that product inhibition of SULTs by the side-product of sulfation reactions, PAP, can occur via the formation of a dead-end enzyme-PAP-substrate complex (120). Because PAP binds to SULT1E1 with an affinity (Kd) of 30 nm (121), this inhibition may be physiologically relevant and can be counteracted by the above-mentioned nucleotide phosphatases that specifically degrade PAP to AMP and phosphate: BPNT1 phosphatase and its Golgi-resident paralog (Golgi-resident PAP phosphatase [gPAPP]) (74). Loss of the *BPNT1* gene leads to impaired protein synthesis resulting in impaired hepatic function and low serum albumin levels in mice (73).

On the other hand, SULT activity is generally regulated by the availability of active sulfate in the form of PAPS (122). PAPS tissue concentrations tend to be in the lower micromolar range (4–80 nmol/g tissue), yet sulfation rates can be relatively high, resulting in depletion of the entire hepatic PAPS pool in less than 1 minute (123), requiring rapid and constant dynamic delivery of PAPS. Biosynthesis of PAPS, on the other hand, is energetically very costly (the three phospho-phospho-bonds that need to be cleaved are equivalent to more than 90 kJ/mol), and hence this pathway and the PAPS synthases involved are subject to tight regulation on various levels, including regulated nucleo-cytoplasmic shuttling (124), dimerization (125), and stabilization by ligand binding (70).

## IV. Cellular Influx and Efflux of Sulfated Steroids

Hydrophilic sulfated steroids require active transmembrane transport for cellular uptake. Because these endobiotics are generally organic anions, cellular influx and efflux are regulated by numerous transporter proteins that belong to two major superfamilies: solute carrier (SLC) transporters, and ATP-binding cassette (ABC) transporters. Evidence suggests that most transporters are bidirectional; however, ABC transporters generally mediate efflux, and SLC transporters mediate influx (126). Two of the 52 gene families within the SLC transporters, the SLCO and the SLC22A superfamilies, contain transporters involved in sulfated steroids transport. The SLCO superfamily contains OATPs (127), and the SLC22A superfamily contains the organic cation transporters and the organic anion transporters (OATs) (128). The OATPs are the primary transporters for sulfated steroid influx, with each OATP possessing distinct uptake kinetics and substrate specificity for different conjugated steroids (Table 3). However, it should be noted that some OATs (OAT1, OAT3, OAT4, and OAT5) can transport sulfated steroids, particularly E_1_S in human placenta (129) and kidney (130).

Conversely, cellular efflux of conjugated steroids occurs through the ABC transporters multidrug-resistant protein (MRP) and in certain instances through breast cancer-resistant protein (BCRP) (131). Usually associated with cancer drug resistance, ABC transporters are transporting polypeptides that utilize ATP-binding and hydrolysis to transport various substrates across membranes. Thirteen MRPs have so far been identified within the human genome, although MRP1 (also known as ABCC1) and MRP4 are considered most efficient in mediating efflux of sulfated steroids.

**Figure 4.**
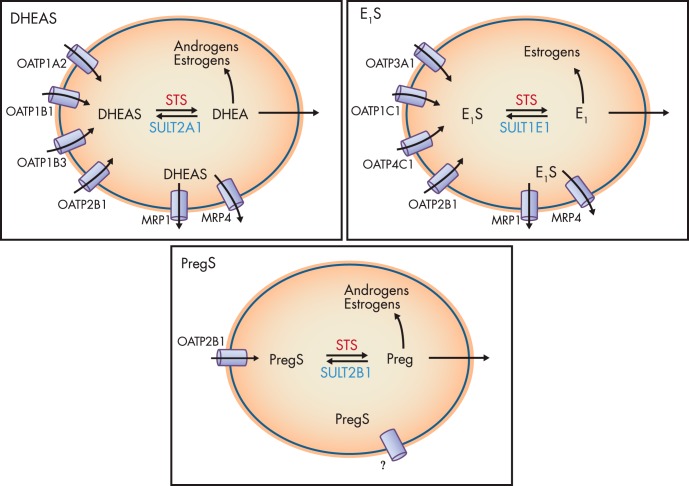
Sulfated steroids are shuttled across the cell membrane by various OATPs. Different OATPs have differing affinities for different steroids. Once intracellular, steroids can be desulfated by STS, and then resulfated by SULTs. The expression ratio between these competing pathways will, most likely, define ultimate sulfation/desulfation outcome. Sulfated steroids can be removed from the cell via MRP1 and MRP4. Nonsulfated steroids act intracellularly, or, because they are lipid soluble, they will diffuse across the cell membrane and potentially act in a paracrine fashion.

Taken together, the relative extent of OATP, MRP, and BCRP tissue expression directly relates to total steroid intracellular concentration, and therefore these transport mechanisms are likely to play key roles in regulating steroid action (Figure 4).

### A. OATP-regulated influx

There are numerous OATPs expressed in almost all epithelia throughout the human body. In addition to conjugated steroids, they are involved in the cellular uptake of a large range of substrates, including bile acids and xenobiotics. The mechanism of OATP-mediated transport remains controversial, although all agree that transport is ATP- and sodium-independent (126). However, what drives uptake is still ill-defined. OATPs can transport bidirectionally, and evidence suggests that they may act as electroneutral exchangers. For example, some OATPs exchange substrates for intracellular bicarbonate (132), glutathione (133), or glutathione conjugates (134). However, transport mechanisms may differ with different OATPs because glutathione does not mediate OATP1B1 and OATP1B3 uptake (135). Furthermore, although acidic pH levels (pH 5.5–6.5) generally elevate OATP2B1-mediated transport (136–139), this is not the case with regard to E_1_S transported by OATP1B1 and OATP1B3 (135). Recent evidence suggests that these two transporters are altered in different ways by both cell membrane potential and local pH conditions (140).

### B. MRP-regulated efflux

The ABC transporter MRP1 was first identified in H69AR cells, a human small cell lung cancer cell line that exhibits resistance to a broad range of natural product-type drugs (141). Along with its role in drug resistance, MRP1 also facilitates efflux of antioxidant glutathione and the proinflammatory leukotriene C4 (142) as well as E_1_S (143) and DHEAS (144), and is expressed in a range of cancerous tissues including hormone-dependent breast (145), prostate (146), and colorectal cancer (147). Transport of E_1_S and DHEAS is distinguished by a dependence on glutathione (148, 149), but the physicochemical properties that determine whether or not sulfated steroid requires glutathione for MRP1-mediated efflux remains unresolved.

However, other MRPs should not be overlooked with regard sulfated-steroid transport. Along with bile acids, MRP8 facilitates the efflux of E_2_17β-glucuronide and E_1_S (150, 151), and it has also been shown to transport DHEAS in the canine kidney cell line MDCK (152). MRP4 has also shown high affinity transport (at 2–10 μm) of DHEAS (149) and therefore may be involved in the regulation of adrenal DHEAS secretion. Intriguingly, Morgan et al (153) demonstrated that MRP4 knockout mice have decreased plasma T concentrations, a process reported to be caused by impaired cAMP-response element-binding protein in Leydig cells. Although these authors measured circulating Androstenedione concentrations, they do not report on circulating DHEAS concentrations in these animals, an experiment that may demonstrate the importance of this MRP4 in adrenal DHEAS secretion.

### C. Estrone sulfate influx and efflux

Most OATP/MRP transport studies have utilized E_1_S because it represents a major substrate for many transport proteins (Table 3). Because estrogens can drive many hormone-dependent cancers, it is not surprising to find that most studies on E_1_S transport are oncologically focused, and little is known about the importance of OATP-mediated uptake in normal physiology. However, studies have shown that many cancerous tissues and cell lines have altered OATP expression compared to healthy tissue. For example, the normally liver-exclusive OATP1B3 is also expressed in gastric, colon, pancreatic, prostate, and breast cancers (154–157).

**Table 3.** OATPs Expression Patterns in Endocrine Tissue and Their Known Conjugated Steroid Substrates

Influx Transporter Expressed in Cell Lines and Tissue (Ref.)	Substrate	K_m_ Values, μm	Refs.
OATP1A2			
Breast (145, 161, 167)	DHEA-3-sulfate	7	173, 175
Prostate (179)	E_2_-17β-glucuronide		424
Placenta (422, 423)	E_1_-3-sulfate	16	167, 173, 425
OATP1B1			
Breast (161)	DHEA-3-sulfate	22	154, 162, 173, 298
Prostate (168)	E_2_-17β-glucuronide	4–24	162, 164, 298
Ovary (426)	E_1_-3-sulfate	0.09–45	162, 163, 173, 298
OATP1B3			
Breast (156, 161)	DHEA-3-sulfate		162, 164, 165, 173, 298
Prostate (157, 168)	E_2_-17β-glucuronide	5–25	162, 164, 298
Placenta (423)	E_1_-3-sulfate		137, 165, 171, 298
Ovary (426)			
Intestine (155, 168)			
OATP1C1			
Testes (166)	E_2_-17β-glucuronide		166
Placenta (427)	E_1_-3-sulfate		166
	T_4_ sulfate		428
OATP2B1			
Breast (161, 166, 429)	DHEA-3-sulfate	9	166
Placenta (181, 430)	E_1_-3-sulfate	1.56–21	137, 138, 163, 166, 172, 173
Intestine (138)	Pregnenolone sulfate		172
OATP3A1			
Breast (161)	E_2_-17β-glucuronide		160
	E_1_-3-sulfate		160

Only six of the 11 OATPs are included because the remaining OATPs have not shown sulfated steroid transport.

Structural investigations of OATP proteins and E_1_S transport are still at an early stage. Transmembrane domains (TMs), essential structural features of membrane proteins critically involved in the proper function of other transporters such as OATs, confer substrate specificity across the OATP family. Thus, it has been shown that TM8 and TM9 in OATP1B1 are critical for its substrate recognition and E_1_S transport (158). More recently, phylogenetic analysis of OATP sequences has revealed that TM2 is also among the TMs that have high amino acid identities within different family members (159). Subsequently, Asp70, Phe73, Glu74, and Gly76 were found to be essential for E_1_S uptake by OATP1B1 (159), although whether this is true across other OATPs remains to be determined.

Initial studies pinpointed hepatic OATP1B1 as the major E_1_S transporter (160), and recent evidence suggests that OATP1B1 is overexpressed in hormone-dependent breast cancer cell lines such as MCF-7 compared to noncancerous epithelial MCF-10A cells (161). Following these early studies, evidence came that OATP1B1 (162, 163), OATP1B3 (162, 164, 165), OATP2B1 (162, 166), and OATP1A2 (167) also transport E_1_S. The expression of these “sulfated-hormone transporters” (OATP1B1, OATP1B3, OATP2B1, and OATP1A2) is low, if not completely absent, in many normal endocrine tissues (166, 168) but is elevated in hormone-dependent cancers arising in these same tissues (168). Indeed, with regard to OATP1B3, there is now strong evidence suggesting that this transport polypeptide becomes a specific cancer-variant isoform localized to colon, lung, and pancreatic cancer (169, 170). This suggests that OATP overexpression and subsequent increased sulfated-hormone cellular influx, along with other substrates, is important in cancer progression, and therefore these proteins represent novel therapeutic targets against estrogen-driven carcinomas. Indeed, inhibiting E_1_S uptake by using organic anions such as bromosulfophthalein, which competes as a substrate for all OATPs, blocks E_1_S MCF-7 cell proliferation (171). Some evidence suggests that it is primarily OATP1B3 that transports E_1_S in breast cancer (156), making it an attractive specific target for inhibitor studies. However, it is evident that many OATPs can transport E_1_S, and thus the jury remains out on whether selectively targeting just one OATP to block E_1_S-uptake is a viable therapeutic strategy.

The kinetics of E_1_S uptake can be influenced by various factors, notably local pH and solute conditions. For example, E_1_S uptake by OATP1B3 is Na^+^ independent (126). Intriguingly, OATP2B1-mediated uptake of E_1_S is enhanced in the presence of progesterone (172, 173). This finding is of special relevance for the formation of estrogens in tissues like placenta and mammary gland, which depend on the uptake of precursor molecules for steroid hormone synthesis like E_1_S and DHEAS, and provides an indication of the importance of OATP transport in normal physiology.

With regard to efflux transport, MRP1 and BCRP both influence total E_1_S uptake. By preloading Caco-2 cells with tritium-labeled E_1_S and then inhibiting BCRP and MRP1 activity, Grandvuinet et al (174) demonstrated that these efflux transporters are actively involved in intracellular E_1_S availability, thus suggesting that the relative expression of OATP, MRP1, and BCRP will ultimately determine intracellular estrogen concentrations. However, definitive studies investigating the relative importance of all these transporters in E_1_S uptake have not yet been performed.

### D. DHEAS influx and efflux

DHEAS transport was first demonstrated in *Xenopus laevis* oocytes overexpressing the human OATP1A2 (175). Similar to most studies on E_1_S, research into DHEAS transport is sparse and again mainly focuses on uptake in cancerous cells. Obviously, interest has focused on the prostate because it is known that prostate cancer cells possess STS activity (176) to desulfate DHEAS, followed by downstream conversion of DHEA to androstenedione (177) resulting in androgen receptor (AR) activation. More pertinently, OATPs involved in DHEAS influx are elevated in human castration-resistant metastatic prostate cancer (178). Indeed, under androgen deprivation, LNCaP cells elevate OATP1A2 expression, and knockdown of this transporter significantly attenuates DHEAS-driven proliferation (179).

In the placenta, DHEAS uptake seems to be regulated by OATP2B1 transport (180). Placental DHEAS uptake correlates with OATP2B1 and BCRP expression, suggesting an interaction of these two proteins in regulating transport of DHEAS (181).

### E. Genetic variation and regulation of OATP expression

The genetic variation in various OATPs (OATP1B3, OATP1B1, OATP1A2) has also been shown to affect overall steroid uptake in a variety of cell lines (182). For example, transfection of SLCO1B1 single nucleotide polypeptide rs4149056 (37041T>C) into HEK293 cells results in lower cell surface expression and thus lower E_1_S uptake compared to wild-type transfections (183). This was also seen with SLCO2B1 SNP rs2306168 (1457C>T) transfection, where E_1_S uptake was less than half that of the wild-type variant (184). Further studies are required to determine whether these SNPs are important in sulfated steroid uptake in cancerous cells.

However, support on the importance of genetic variation in OATPs and DHEAS uptake comes from various clinical studies examining these transporters and prostate cancer outcomes. For example, in a cohort of 538 patients suffering metastatic hormone-sensitive prostate cancer, men with each of three OATP2B1 alleles (rs12422149 [935G>A; Arg312Gln], rs1789693, and rs1077858) had a shorter median time to progression of 10, 7, and 12 months, respectively; and this effect was additive (185). Patients with multiple “at-risk” OATP2B1 variants (including OATP2B1 allele rs12422149 935G, which has a high-transport efficiency for DHEAS), who also had the high T transport OATP1B3 SNPs, had the shortest time to progression. These data have been supported by a study examining 532 Japanese men, where homozygosity for the OATP2B1 rs12422149 935G variant was associated with shorter median time to progression (186).

Little is known regarding OATP regulation, and we will only focus on the OATPs with substrate affinity with conjugated steroids. Generally, OATP expression is controlled by transcriptional regulation (126) and is most likely tissue specific. OATP1B1 expression is dependent on Hepatic Nuclear Factor α1 (187, 188) and may also involve Signal Transducer and Activator of Transcription 5 (189), Interferon-γ (190), and IL-1β (191). In contrast, it is bile acids that can up-regulate OATP1A2 expression in intestinal and liver tissue (192), although in breast tissue OATP1A2 regulation is significantly associated with PXR expression (193). Meyer zu Schwabedissen et al(167) have also demonstrated that OATP1A2 is up-regulated in malignant breast tissue, with this elevation directly related to E_1_S uptake. Furthermore, OATP1A2 expression is regulated by activation of the nuclear receptor PXR, whose primary function is to sense foreign toxins and in response up-regulate OATPs for detoxification and clearance purposes.

## V. Disease-Causing Mutations Affecting Steroid Sulfation and Desulfation

### A. Pathogenic mutations in steroid sulfatases and SUMF1

#### 1. X-linked ichthyosis (STS deficiency)

Mutations or deletions of the *STS* gene result in a skin condition called “X-linked ichthyosis” (XLI), which in approximately 80% of cases is due to complete deletions of the *STS* gene (31, 194, 195). XLI is also termed STS deficiency and represents one of the common inherited metabolic disorders, with 1:6000 live births and no geographical or ethnical variation (196–198).

Generally, ichthyosis refers to genetically and acquired disorders of the skin characterized by abnormal keratinization; the skin often resembles “fish scales,” explaining the origin of the term ichthyosis from Greek *ichthys*, translated as *fish*. XLI was first recognized in the 1960s as a distinct form of ichthyosis due to a distinct clinical appearance and the mode of inheritance (196, 199). It is characterized by large, dark-brown, and tightly adherent scales found at most areas of the skin, but predominantly symmetrically located on the trunk, the neck, and the extensor surfaces. The scalp is nearly always affected; however, plantar and palmar surfaces are spared. The scaling starts a few months after birth, and generally tends to improve during the summer months.

The underlying pathophysiology of the excessive scaling/hyperkeratosis results from impaired cholesterol metabolism. STS catalyzes the breakdown of cholesterol sulfate in the outer layers of the skin (stratum granulosum and stratum corneum) (200). In patients with XLI, where there is no STS activity, this breakdown is impeded and cholesterol sulfate, which physiologically stabilizes cell membranes and adds cohesion (201), accumulates in the stratum corneum causing partial retention hyperkeratosis with visible scaling (194, 200, 202).

Cryptorchidism has been reported in up to 20% of patients with XLI (203–207). Because the patients from these reported case series were not genetically characterized, it is unclear whether the testicular maldescent is a direct consequence of STS deficiency or secondary to deletions of adjacent genes to the *STS* locus. Indeed, complex syndromes including XLI due to contiguous gene deletions of the X chromosome are reported, including Conradi-Hunermann syndrome (OMIM 302960; limb shortening, epiphyseal stippling, craniofacial defects, short stature) and Rud syndrome (OMIM 308200; cryptorchidism, retinitis pigmentosa, epilepsy, and mental retardation). Lynch et al (208) reported an X-linked recessive pattern of concomitant XLI with hypogonadism in one family with five males affected. Although anosmia has not been reported in this kindred, it seems likely that a contiguous gene syndrome affected both the STS and KAL1 loci. Recent investigations in a fully genetically characterized cohort of XLI patients and genetic abnormalities confined to the *STS* gene indicate that testicular maldescent is rare. Of 30 males with XLI, only one boy had unilateral cryptorchidism (unpublished data), which is within the range of the general population risk in Western countries (209).

An association between STS deficiency and testicular cancer independent of testicular maldescent has been hypothesized and reported in two patients with XLI (210); however, this report is the only one published to date. The very first clinical presentation of XLI may occur at birth because efficient desulfation of DHEAS and consequent conversion of DHEA to estrogens is important for cervical softening (211), which would be disrupted in STS deficiency. Thus, women carrying children affected by XLI have reported prolonged labor due to insufficient cervix dilatation (cervical dystocia) (204, 212, 213)—a severe and unexpected birth complication where perinatal death has been reported (214). Prenatal diagnosis of STS deficiency is possible because maternal estrogen excretion is decreased, and hence characteristically low estriol is found. GC-MS analysis of maternal urine can help to distinguish fetal STS deficiency from other conditions associated with low estriol, such as aromatase deficiency or congenital adrenal hyperplasia due to P450 oxidoreductase deficiency, because sex steroid precursor metabolite excretion in maternal urine during a pregnancy affected by XLI is normal (215–217).

Androgen metabolism has been studied in several cohorts of male XLI patients (218–222). Interestingly, increased serum DHEAS was not consistently found in XLI/STS-deficiency patients. Lykkesfeldt et al (221) investigated 20 adult males with XLI and found decreased downstream androgens with a trend toward higher serum DHEAS and lower serum androstenedione levels. An in vivo study in healthy young men investigating DHEA-DHEAS interconversion suggests that DHEA sulfation is the predominant direction, whereas desulfation by STS does not seem to play a role in normal adult physiology, with no increase in circulating levels of DHEA or sex steroids after iv DHEAS administration (223). This is confirmed for adult males from our cohort of 30 mixed adult and pediatric patients with STS deficiency and age-matched controls; however, the ratio of serum DHEA/DHEAS, reflecting in vivo STS activity, is increased in the prepubertal healthy boys, suggesting that STS is active before puberty, contributing toward peripheral androgen activation. In addition, the global 5α-reductase activity, determined by urinary steroid profiling, is increased in STS-deficient males, indicative of a compensatory mechanism counteracting a relatively reduced rate of tissue-specific androgen activation (unpublished data).

Although STS may not contribute to peripheral androgen activation in healthy male adults, ample placental STS activity during pregnancy substantially increases circulating DHEA and sex steroid levels; accordingly, increased levels after iv DHEAS challenge have been described (224).

#### 2. Multiple sulfatase deficiency

Multiple sulfatase deficiency (MSD; OMIM 272200) is a rare and severe autosomal recessive disease that affects the function of all sulfatase enzymes, leading to a rather complex phenotype, which essentially incorporates the features of each single known sulfatase deficiency. The elucidation of the underlying pathology in patients with MSD has led to the discovery of a unique post-translational event, which is shared by all human sulfatase enzymes: the activation of a cysteine residue to form an activated FGly at the active site of the sulfatase, which is thought to attack and subsequently cleave the sulfate moiety off the substrate (28) (see *Section II.A,1*). In 2003, the *SUMF1* gene was discovered to encode the FGE, revealing the molecular basis of MSD (40, 41). To date, there are about 30 mutations of the *SUMF1* gene reported in patients with MSD, and clear genotype-phenotype correlations have been observed linked to the residual activity of FGE (225), leading to manifestations with severe neonatal, late infantile, or rarer mild juvenile forms of MSD (226, 227).

To further understand the pathology of SUMF1 deficiency, various groups have identified eight other disorders genetically and clinically linked to deficiencies of distinct human sulfatase enzymes. Six of them represent lysosomal storage disorders, where the sulfatase enzyme fails to exert its catabolic function such as the desulfation of sulfated glycolipids (via arylsulfatase A), leading to the accumulation of sulfatides and the progressive demyelinization observed in metachromatic leukodystrophy (OMIM 250100); or the accumulation of GAGs, including heparin sulfate, dermatan sulfate, keratin sulfate, and chondroitin sulfate, as observed in the various types of mucopolysaccharidosis (see Ref. 14 for excellent review and *Section V.B.1*). Patients with MSD therefore show severe neurodegeneration with mental retardation, hepatosplenomegaly, short stature (resembling mucopolysaccharidosis), combined with XLI-type skin and skeletal changes as observed in chondrodysplasia punctata (OMIM 302950) (227).

#### 3. Autism and ADHD

Recent studies have shown an association of XLI with behavioral disorders, including autism, attention deficit-hyperactivity disorder (ADHD), and social communication deficits; however, in the affected subjects, large gene deletions in the proximity of the STS locus have been found that included the *NLGN4* gene encoding neuroligin 4, a synaptic peptide that has been previously implicated in X-linked autism and mental retardation (228). However, the *STS* gene in 384 patients with ADHD identified two SNPs of the *STS* gene that were significantly associated with ADHD (229). The authors hypothesized that disturbed neuronal DHEA-DHEAS metabolism might result in altered neurotransmitter function contributing to the observed behavioral abnormalities. This has been supported in STS knockout mice that develop attention disorders consistent with ADHD (230), which can be alleviated with the administration of DHEAS (231).

### B. Pathogenic mutations in steroid sulfotransferases and PAPS synthases

#### 1. Bone and cartilage malformations

Inborn defects in various genes involved in sulfate uptake, activation, and utilization have been linked to developmental defects in cartilage and bone (232). Diminished sulfate uptake is caused by mutations in the diastrophic dysplasia sulfate transporter gene (*SLC26A2*) and causes diastrophic dysplasia, achondrogenesis type IB, atelosteogenesis type II, and a recessive form of multiple epiphyseal dysplasia (68).

A missense mutation in the gene encoding the sulfate-activating enzyme PAPSS2 has been described as associated with a brachymorphic phenotype in mice (233), with normal levels of GAGs that are, however, severely undersulfated (234). Human PAPSS2 mutations were first described in the context of a severely affected consanguineous Pakistani kindred (235, 236). Mutations in *PAPSS2* can cause varying forms of bone malformation in humans, ranging from subclinical brachyolmia with only mild radiological spinal changes (237), via overt brachyolmia with dysplasia confined to the spine (15 reported cases so far) or with additional minimal epimetaphyseal changes only visible on x-ray (four cases), to overt spondyloepimetaphyseal dysplasia with both vertebrae and long bones affected (23 reported cases), as summarized recently (238).

Undersulfation of the GAG chondroitin sulfate may also be caused by inactivating mutations of the chondroitin 6-O-sulfotransferase gene, *CHST3*, resulting in severe chondrodysplasia with progressive spinal involvement (239) and congenital joint dislocations in humans (240). It has been assumed previously that undersulfation of GAGs directly leads to changes in the biomechanical properties of cartilage (105). However, more likely, morphogen signaling involving hedgehog proteins, wingless-related proteins, and fibroblast growth factors may be compromised by changed chondroitin sulfate proteoglycans because all of these growth factors interact with the extracellular matrix (241).

Bone and cartilage malformation caused by sulfation defects contrasts with bone and cartilage phenotypes due to sulfatase defects. The sulfate group transferred to N-acetylgalactosamine of chondroitin sulfate by CHST3 is the same as that removed in the lysosomes by Gal-NAc-6-sulfatase, the enzyme deficient in mucopolysaccharidosis type IV A (also known as Morquio syndrome; OMIM 253000). This highlights the importance of the correct balance of sulfation and desulfation for bone and joint development in humans.

Furthermore, the side-product of sulfation reactions, the bis-phospho-nucleotide PAP, also has an impact on bone development. The phosphatase gene *BPNT1*, responsible for removal of cytoplasmic PAP, has a paralog localized to the Golgi compartment, gPAPP (74), and this gene has been associated with impairment of skeletal development (242). More recently, patients were described with homozygous missense (243) and homozygous truncation mutations (244) in the gene encoding gPAPP. Affected patients presented with short stature, joint dislocations, brachydactyly, and cleft palate; these phenotypes highlight the importance of fully functional sulfation pathways in the development of skeletal elements and joints.

#### 2. Androgen excess, PCOS, and metabolic disease

Androgen excess is one of three hallmarks of polycystic ovary syndrome (PCOS), the most common female endocrine disorder, affecting about 6–9% of women worldwide (245). Furthermore, increased androgen levels are associated with an adverse metabolic phenotype, increasing the risk of insulin resistance, type 2 diabetes, obesity, and cardiovascular disease (246). Many molecular causes for androgen excess exist, with one possibility a failure in the sulfation pathway that converts DHEA to DHEAS, the most abundant steroid in the human circulation. The obvious candidate gene for such a disorder, SULT2A1, has indeed been suggested to play a role in inherited androgen excess in PCOS (247). Two recent studies looked at the association of common genetic variants (minor allele frequency > 5%) in SULT2A1 and PAPSS2 with androgen status without an obvious link between inherited genetic variation and androgen excess (248, 249). However, rare inactivating genetic variants of the *PAPSS2* gene result in apparent SULT2A1 deficiency associated with androgen excess. This results from decreased conversion of DHEA to DHEAS, consequently increasing the DHEA pool available for downstream conversion to active androgens. The resulting clinical androgen excess manifests with premature pubarche and early-onset PCOS, and of note, in both families that were characterized in detail (237, 238, 250), the heterozygous mothers carrying a major loss-of-function mutation on only one allele clinically presented with PCOS. An association of circulating DHEAS levels with common variants in the *SULT2A1* and *PAPSS2* genes has been recently excluded in a population-based study (249). Additionally, in a large PCOS cohort study (248), common SULT2A1 and PAPSS2 variants did not present as risk alleles, although a common SULT2A1 allele variant was associated with the serum DHEA/DHEAS ratio. Further studies in PCOS cohorts including analysis of rarer genetic variants are warranted.

Obesity is an important risk factor for PCOS because it contributes further to the characteristically decreased insulin sensitivity. Circulating estrogen levels may be increased in obese patients due to enhanced aromatization within adipose tissues (251), and estrogens can regulate fat mass distribution and glucose metabolism. Thus, estrogen action in obesity will be regulated by steroid sulfation because the estrogen sulfotransferase SULT1E1 is highly expressed in adipose tissue of male mice and induced by T in female mice (252). Overexpression of SULT1E1 in a murine transgenic model results in reduced parametrial and sc inguinal adipose mass and reduced adipocyte size, but normal retroperitoneal and brown adipose deposits (253); SULT1E1 overexpression also prevents adipocyte differentiation (254). In humans, however, SULT1E1 is a proadipogenic factor (252). Its expression is reported to be low in preadipocytes but increases upon differentiation to mature adipocytes. Overexpression and knockdown of SULT1E1 in human primary adipose-derived stem cells promotes and inhibits differentiation, respectively (252). If this holds true, SULT1E1 could represent a drugable target, and adipose-specific SULT1E1 inhibitors could be used to inhibit the turnover of adipocytes in obese patients.

Steroid sulfation and desulfation pathways have both been implicated in improving and/or worsening metabolic outcomes associated with obesity and type-2 diabetes. Estrogen and androgen concentrations have been implicated in regulating energy and glucose homeostasis. For example, mice lacking the aromatase enzyme become obese due to attenuated physical activity and decreased lean body mass (255), and ERα-deficient mice exhibit reduced energy expenditure leading to an obese phenotype (256). Estrogen deficiencies also result in impaired insulin sensitivity in both aromatase knockout (255) and ERα knockout mice (257). Conversely, estrogen administration improves insulin sensitivity in high-fat-diet female mice (258) and ob/ob obese mice (259).

This evidence suggests an importance in both STS and SULT1E1 activity in improving metabolic outcomes associated with obesity. Recent studies have examined the effect of both enzymes on metabolic function in obesity and diet-induced type 2 diabetes in mice. Hepatic SULT1E1 expression, although normally low, is elevated in type 2 diabetic mice, and loss of SULT1E1 improved metabolic function in these same animals (260). Furthermore, SULT1E1 ablation increased energy expenditure and insulin sensitivity and decreased hepatic gluconeogenesis and lipogenesis. This metabolic benefit resulted from decreased estrogen sulfation, and therefore an increased estrogenic activity in the liver; this effect was not seen in ovariectomized mice (260). The same group then developed a liver-specific STS knock-in mouse model and demonstrated that increased hepatic active estrogen concentrations are associated with an improved metabolic function when compared to obese and type 2 diabetic animals. Furthermore, they show that hepatic STS activity is increased in mice given high-fat diets and in ob/ob obese animals (261). This suggests that SULT1E1 and STS activities are important in energy homeostasis and that up-regulation of STS and thus an increased synthesis of estrogens may be a hepatic defensive response against the metabolic syndrome.

Intracellular accumulation of lipids, inflammatory responses, and subsequent apoptosis are major pathogenic events of metabolic disorders. Sulfated oxysterols also play a role in lipid metabolism and obesity. For a long time, it has been known that oxysterols, derivatives of cholesterol, bind to LXR nuclear receptors and up-regulate hepatic de novo lipogenesis (262). LXR activation also prevents bile acid toxicity (263). On the other hand, LXR expression correlates with intrahepatic inflammation and fibrosis in patients with nonalcoholic fatty liver disease (264). Recently, it became apparent that these nuclear receptor ligands, when sulfated, are not merely blocked from binding, but are actively inhibiting nuclear receptor signaling by yet unknown mechanisms (265), putting steroid sulfotransferases into the context of energy metabolism and regulation. Furthermore, sulfated sterol signaling is not limited to LXR receptors, but it affects several other members of the nuclear receptor family acting then as metabolic sensors of intracellular lipid, bile acids, and cholesterol levels: CAR, farnesoid X receptor, peroxisome proliferation activator receptors, and retinoid X receptor (266). Sulfation of bile acids and oxysterols is catalyzed exclusively by the SULT2A and SULT2B enzymes (100, 267). Hence, sulfated oxysterols may represent candidates for the development of novel therapeutic approaches to nonalcoholic fatty liver disease (268), a metabolic complication of obesity that continues to increase in prevalence, now representing the second most common cause of liver transplantation.

## VI. Dysregulation of Steroid Sulfation and Desulfation

### A. Cancer

**Figure 5.**
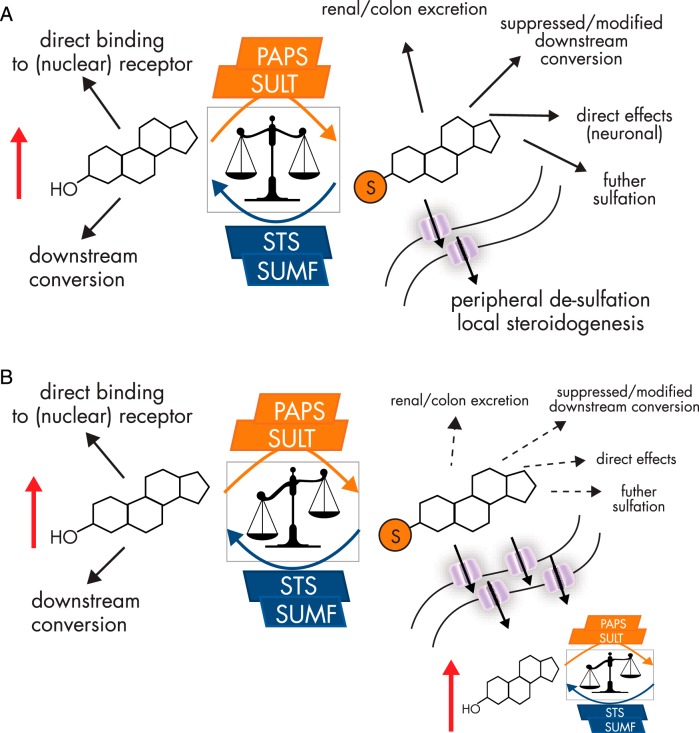
A, The balance between sulfation and desulfation strongly influences steroid hormone action. The nonsulfated steroid may exert its biological effect by binding to its cognate nuclear receptor or may be downstream converted to more active steroids. Once sulfation occurs by one of various sulfotransferases, solubility of the steroid is dramatically increased, facilitating renal excretion, but also circulatory transit fueling peripheral desulfation and local steroidogenesis. Sulfation may also suppress or modify downstream conversion by masking one of several functional groups; further sulfation steps may occur or sulfated steroids may exert biological effects directly. B, Dysregulation of sulfation and desulfation pathways dramatically alters available active steroids. In disease, especially in cancer, SULT enzymes expression and thus activity are decreased, whereas STS activity is elevated. This situation favors desulfation and therefore results in an elevated local synthesis of active steroids. Furthermore, OATP expression is also elevated in many cancers, increasing the intracellular availability of sulfated steroids to STS action.

Steroid metabolism is significantly altered in many endocrine-related cancers (269). Evidence suggests that sulfation pathways are down-regulated, whereas STS activity increases in many tumors, thus favoring desulfation and therefore downstream conversion of steroids into more active metabolites (Figure 5).

#### 1. Breast

Most breast cancers are initially estrogen responsive and exhibit increased intratumoral estrogen concentrations compared to adjacent normal breast tissue (270). Hence, it is of interest that the highest incidence of breast cancer is observed in postmenopausal women despite cessation of ovarian estrogen synthesis and the consequent drop in circulating estrogen concentrations. Estrogens can still be produced in postmenopausal women by tissue-specific local conversion of androstenedione to E_1_, and to a lesser extent T to E_2_, by aromatase (271). However, estrogens are sulfated by E_1_ sulfotransferase (SULT1E1) and phenol sulfotransferase (SULT1A1), and this accounts for the high circulating E_1_S concentrations observed in postmenopausal women, with this E_1_S pool acting as a reservoir for peripheral conversion to E_1_ by STS (35).

Significant scientific discussion surrounds the relative importance of the two primary pathways for active estrogen generation, E_1_S desulfation, and androgen aromatization in hormone-dependent breast cancer. Whereas increased aromatase protein expression parallels increased intratumoral E_2_ concentrations (272), there is currently limited support for STS expression directly correlating with locally increased E_2_ concentrations. However, STS activity can be 50–200 times higher than aromatase activity in breast cancer tissue (273), and STS mRNA is frequently detected in breast tumors, whereas aromatase levels are relatively low (274). This suggests that STS, rather than aromatase, may be the primary driver for local E_1_ production in hormone-dependent breast cancer (275, 276). Enzyme kinetic studies show that STS activity is higher than aromatase not only in cancerous tissue but also in normal breast (270). In addition to local estrogen metabolism via STS and aromatase, serum estrogen levels for E_1_, E_1_S, E_2_, and E_2_ sulfate (E_2_S) have been reported to fall after surgical removal of STS-positive breast cancer in postmenopausal women, implying an additional systemic effect and indicative of the importance of STS activity in forming active estrogens (17, 277).

In breast cancer, STS mRNA expression (278) and activity (275) are higher in cancerous compared to normal breast tissue, with elevated STS mRNA expression being significantly associated with lymph node metastasis, histological tumor grade (279), and poor prognosis (280). Soft tissue breast cancer metastasis expresses higher STS mRNA compared to primary tumors (281). Furthermore, SULT1E1 expression, responsible for E_1_ sulfation, is decreased in breast cancer, with an inverse correlation between tumor histological grade and levels of intratumoral SULT1E1 immunoreactivity (17, 282, 283). Thus, it is possible that breast cancers favor local desulfation pathways to increase E_1_ availability from high circulating E_1_S. Subsequent E_1_ conversion, by 17βHSDs (17βHSD-1), potentially results in E_2_ concentrations that are considerably higher in breast cancer tissue compared to circulating levels (284). Intriguingly, patients treated with the aromatase inhibitor exemestane have elevated breast tumor STS and 17βHSD-1 immunoreactivity, which both correlate negatively with tumor Ki67 proliferation index (285). This suggests a compensatory mechanism via E_1_S desulfation in response to local E_2_ depletion caused by aromatase inhibition.

Surprisingly, however, there are no definitive studies correlating breast intratumoral E_1_ and E_2_ concentration and STS activity and expression. Haynes et al (286) have shown that STS mRNA may be down-regulated in breast cancer from both premenopausal and postmenopausal women compared to matched controls. Furthermore, they suggest that no correlation was observed between intratumoral E_2_ and STS mRNA expression, and there is limited evidence to support a role for STS in establishing intratumoral E_2_ levels in these patients. However, they failed to examine STS activity in these tissue samples, and it is thought that post-translational modification of the STS enzyme is more important in determining STS activity than measuring STS mRNA expression levels alone (61). Furthermore, these results are in sharp contrast to other findings that show breast cancer patients have a significantly longer disease-free survival if their STS mRNA levels are low (278) and that STS protein expression correlates with ERα expression (287). Also, STS activity has consistently been shown to be elevated in breast cancer tissue (57, 269, 278, 288).

The regulatory mechanisms underlying increased STS expression in breast cancer are not fully understood. Current evidence suggests that inflammatory cytokines, TNFα and IL-6, increase STS activity (57, 61), although this has been disputed by a study showing negative correlation between TNFα/IL-6 expression and STS expression in soft tissue breast cancer metastases and primary tumors (281). Expression of tissue-specific transcripts of STS may also be controlled by ERα signaling in normal and cancerous breast tissue (52); these studies also demonstrated that ERα-positive human breast cancer tissue expresses more active STS isoforms that are up-regulated by local E_2_ concentrations, thus promoting cancer progression (52). Supporting this, a recent study investigating 45 primary breast tumors showed that STS and 17βHSD-1 expression correlates with ER activity, as measured by transfection using adenovirus vectors carrying an ERE-tk-GFP reporter gene (287). Thus, ERα activation is important in regulating STS activity and subsequent E_1_ and E_2_ synthesis, although a full understanding of what regulates STS and SULT1E1 expression and activity in breast cancer remains to be elucidated.

But what of DHEAS desulfation and the subsequent synthesis of T and dihydrotestosterone (DHT) in breast cancer? Before aromatase action, desulfation of DHEAS by STS generates androgens, and although androgens can act as estrogen prohormones, they themselves may have a role in breast cancer incidence, risk, and proliferation (282, 289). Historically, androgens were given therapeutically to breast cancer patients (290, 291) to improve survival outcomes. However, patients suffered undesirable side effects such as hirsutism and amenorrhea.

Currently, controversy exists as to the significance of androgenic effects in breast cancer, and therefore, by extension, the importance of local DHEA sulfation and desulfation. Unlike estrogens, in normal breast androgens inhibit proliferation (292, 293). However, in breast cancer, androgenic effects are complex and most likely depend on the differing intracrinology of different breast carcinomas (see Ref. 294 for excellent review). A recent systematic review exploring 19 studies with a total of 7693 women found AR expression in 60.5% of breast cancers. AR expression was more common in ERα-positive tumors (74.8%) compared to ER-negative (31.8%), and patients expressing AR had improved overall survival (295). This would support the rationale for selective AR activation as a potentially attractive therapeutic approach for breast cancer.

Although circulating DHEAS concentration correlates positively with breast cancer incidence in premenopausal (296, 297) and postmenopausal women (298, 299), the importance of androgen synthesis through DHEAS desulfation via STS in breast cancer has not yet been fully explored. Early studies showed that DHEAS caused proliferation in T47D breast cancer cells, known to have STS activity (300), even when cotreated with tamoxifen, implying that androgens influence breast cancer proliferation through AR activation (301) and not just through estrogenic metabolites (302). However, other studies contest these facts, with some showing DHEA as antiproliferative in MCF-7 (303) but not in MDA-MB-231 or Hs578T cells (304).

In vitro (305) and in vivo (306) studies using STS inhibitors imply that the dominant effect of increased STS activity in breast cancer is not inhibition of growth by androgens, but rather estrogen-driven proliferation. However, phase I clinical trials of Irosustat (STX64, 667Coumate), a potent STS inhibitor (307), in breast cancer patients demonstrated that blocking STS activity not only significantly reduced circulating E_1_S, but also lowered plasma DHEA and androstenedione concentrations, and if DHEA is indeed antiproliferative in breast cancer, this may have unwanted consequences for this treatment approach.

#### 2. Prostate

In men, the prostate is the major peripheral tissue where STS activity contributes to the local synthesis of biologically active androgens. Unlike breast cancer, where a higher exposure to estrogens is associated with increased malignancy risk, prostate cancer incidence is not associated with high circulating androgen concentrations (308). Men with prostate cancer, who have been treated by castration, can be successfully treated further by adrenalectomy (309). Although outdated, this approach works because the adrenals secrete DHEAS, which can be activated to the active androgens T and DHT in prostate tissue (310).

Similar to breast cancer, STS activity has been detected in normal (311) and cancerous (312) prostate tissues. Furthermore, SULT1E1 (17) and SULT2B1 (313) mRNA are also detected. The expression patterns of these enzymes will therefore influence local estrogen and androgen synthesis. The prostate cancer cell line LNCaP exhibits higher STS activity than some breast cancer cell lines (176). STS activity is also present in DU-145 and PC-3 prostate cancer cells and in human prostate cancer biopsies (312). DHEAS can be metabolized to DHEA in these cells, with this hydrolysis being blocked by the STS inhibitor oestrone-3-O-sulphamate (314). DHEA inhibits, whereas T induces, apoptosis in LNCaP cells under serum-deprived conditions (315); this effect may be due to differing binding affinities to the AR of these two steroids, leading to different coactivator/corepressor recruitment. With regard to proliferation, administration of DHEAS to castrated male rats increases ventral prostate and seminal vesicle weights and increases circulating DHEA and DHT concentrations, with this effect abolished by STS inhibition (316). However, DHEA alone has little effect on LNCaP or LAPC-4 growth, unless they are cocultured with prostate stromal cells (317, 318), suggesting that downstream androgen biosynthesis from DHEA requires both prostate stromal and epithelial components. Intriguingly, in prostate cancer patients treated with the nonspecific P450c17 inhibitor, ketoconazole, or the specific P450c17 inhibitor, abiraterone acetate, significant (∼20 μg/dL) circulating DHEAS concentrations were still present, suggesting that this could act as a depot for further downstream androgen formation via desulfation and AKR1C3 action (319). Furthermore, a reasonably substantial (2.0–2.5 ng/mL) concentration of DHTS circulates in men (320) and, similarly to E_1_S in women, could act as a reservoir for peripheral DHT synthesis. Indeed, prostate cancer patients exhibit significantly elevated circulating DHT and DHTS concentrations compared to aged-matched controls (321), suggesting their importance in this malignancy's development and a potential further role for STS in active androgen formation.

Recently, a role for estrogen signaling in prostate cancer development, particularly through ERβ splice variants, has also been postulated (322), and evidence is growing that ERβ may modulate androgen action and therefore prostate cancer development (323). Men have significant E_1_S concentrations in circulation (see Table 1). STS activity is present in healthy and malignant prostate tissue (312), and prostatic E_1_S uptake may increase during aging (324). Furthermore, circulating E_2_ concentrations are elevated in patients with prostate cancer (325), suggesting estrogenic influences on the incidence and development of this malignancy.

Interestingly, SULT1B1, a sulfotransferase that can sulfate DHEA, is down-regulated in prostate cancer compared to normal prostatic tissue (108). Knockdown of SULT1B1 in LNCaP cells increases DHEA-induced proliferation (326), implying that the STS/SULT1B1 ratio in the prostate regulates DHEAS/DHEA-induced proliferation. This ratio is likely to be influenced by local inflammatory conditions, as shown by Suh et al (59) who assessed whether TNFα can induce STS expression; LNCap and PC-3 cells up-regulated STS expression in a TNFα concentration and time-dependent manner. They further demonstrated that at least part of this effect was via the phosphatidylinositol 3 (PI3)-kinase/Akt pathway because PI3-kinase inhibitors and AKT inhibitors suppressed STS mRNA up-regulation induced by TNFα. The same group later examined PC-3 cells and found that IGF-2 increased STS expression via the same PI3-kinase/Akt pathway (327).

The fact that inflammation and cancer are often seen together (328), with evidence linking prostatitis with prostate cancer risk (329) and high TNFα associated with poorer prognosis with earlier onset of castration-resistant prostate cancer (330), it is interesting to surmise that local inflammatory conditions may impact on the balance of sulfation and desulfation in prostate tissue to drive proliferation. Intraprostatic hormonal dysregulation occurs in benign prostate hyperplasia (BPH) with an increase in active sex steroids. STS activity and tissue concentrations of DHEA and E_1_ were found to be higher in BPH tissue compared to circulating concentrations (331, 332). However, clinical evidence of an association between TNFα, DHEAS, and DHEA concentrations, and BPH and prostate cancer progression is currently lacking.

#### 3. Endometrium

Endometriosis is a common gynecological condition defined as proliferation of ectopic endometrial tissue and stroma, ie, in locations other than the uterus. It is associated with pelvic pain, dyspareunia, dysmenorrhea, and infertility. Endometriosis is estrogen-dependent and therefore occurs in women of reproductive years (333). The premenopausal endometrium undergoes a regular and predictable sequence of proliferation and secretion followed by menstruation. STS has been shown to have a cyclical change in activity during the menstrual cycle, suggesting that, in this tissue at least, it is regulated by hormonal factors as well as regulating local estrogen and androgen synthesis (334). In human endometrial tissue, STS activity peaks at the early secretory stage and declines thereafter (335). IL-1β, known to increase at the secretory phase of menstruation, suppresses STS mRNA and activity in human endometrial stromal cells (336). STS activity is also elevated in ovarian and rectovaginal endometriosis compared to disease-free endometrium with enzyme ratios (STS/SULT1E1 and HSD17B1/HSD17B2), favoring E_2_ production (337). Indeed, SULT1E1 protein has been shown to be down-regulated in human endometriosis tissue (338), and increasing STS activity correlates with disease severity (339). Not all studies have shown this correlation, but STS activity is consistently high in eutopic and ectopic endometrial tissue (340). STS inhibitors reduce STS activity in endometriotic implants (341), and inhibition of STS in murine models of endometriosis decreases disease severity (342). Interestingly, randomized, double-blind, placebo-controlled trials examining combining E2MATE, an STS inhibitor, with norethindrone acetate, a synthetic progestin, demonstrated a synergistic effect on STS inhibition, suggesting this approach as a potential treatment option for endometriosis patients (343).

Increased STS activity and expression are also associated with endometrial cancer. Both nuclear ERs are expressed in the endometrium, with ERα more highly expressed than ERβ. Data on ER expression alterations in both endometriosis and endometrial cancer are conflicting (344–346). However, as with breast and colorectal cancer, estrogen levels have been shown to be higher in endometrial tumor tissue compared to normal, with E_2_ tissue levels correlating positively with disease stage and tumor invasion (347). Prolonged lifetime estrogen exposure and reproductive factors such as early menarche, nulliparity, and late menopause increases the risk of endometrial cancer (348–350). Hormone replacement therapy (HRT) can increase the risk of endometrial cancer because estrogens stimulate proliferation in the endometrium, unless it is combined with progesterone therapy, as this hormone differentiates endometrial cells.

Despite endometrial cancer being estrogen driven, paradoxically and similar to breast cancer, the greatest incidence is in postmenopausal women (351), again indicating peripheral estrogen synthesis. Although aromatase activity is not present in endometrial tissue (352), STS activity is increased up to 12-fold in human endometrial cancer tissue (353, 354). Utsunomiya et al (355) found 86% of endometrial tumors immunoreactive for STS and 29% for SULT1E1. The STS/SULT1E1 ratio correlated with poorer prognosis, with a higher ratio associated with high circulating E_2_ levels. Of note, Lukanova et al (349) showed that elevated circulating estrogens and androgens were associated with endometrial cancer risk. They hypothesized that although serum androstenedione and T positively correlated with endometrial cancer risk, it cannot be concluded whether this is mediated primarily through estrogen conversion or by AR activation. Thus, attenuating both estrogenic and androgenic sex steroids through STS inhibition appears to be a feasible therapeutic strategy in endometrial cancer.

The endometrial cancer cell lines Ishikawa, HEC-1A, HEC-1B, and RL-95 do not metabolize androstenedione to E_1_ or E_2_, suggesting that aromatase is not important in these cells (356). However, E_1_S is hydrolyzed in these cells, albeit at a low rate, and an in vivo Ishikawa xenograft model in mice has demonstrated that endometrial cancer proliferation can be driven by E_1_S and inhibited by STS inhibitors Irosustat and STX213 (357). Unfortunately, phase II trials of Irosustat as a monotherapy in endometrial cancer patients were discontinued in 2011 after data indicated no beneficial effect of STS inhibition when compared to megestrol acetate. However, future studies will examine the effects of combining STS inhibition with standard treatment options for endometrial cancer patients.

#### 4. Colorectal

Colorectal cancer (CRC) is not routinely referred to as hormone sensitive; however, estrogens and androgens are implicated in both normal gastrointestinal physiology and carcinogenesis (358). Evidence supports a role for estrogens not only in CRC pathogenesis, but also in protection. This dual role of active estrogens was first postulated from the Women's Health Initiative (WHI) study that demonstrated combination (equine E_1_S plus progestins) HRT resulted in 40% CRC risk reduction, suggesting that estrogen or progestins may have protective roles. The combined oral contraceptive pill also reduced CRC risk by 20% (359). However, women diagnosed with CRC while using HRT had higher tumor grades, suggesting either that HRT delayed clinical diagnosis or that estrogens also play a role in tumor progression (360). A large study by Zervoudakis et al (361) explored the association between lifetime endogenous estrogen and CRC, finding that higher exposure increased risk in postmenopausal women. Contradictorily, as a population, males are at an increased risk of CRC, in particular compared to premenopausal women. Younger women also have an improved survival (362), suggesting that the relationship between estrogens and CRC incidence is complex.

Estrogen concentrations, as measured by LC-MS, are higher in human CRC tissue compared to normal colonic mucosa, and when separated into E_1_ and E_2_, E_1_ concentrations predominated (363), suggesting high CRC intratumoral E_1_S desulfation. High local total estrogen (E_1_ and E_2_) concentrations are associated with reduced CRC survival (337). Interestingly, estrogen concentrations are concordant with high STS and low SULT1E1 expression rather than aromatase, and the STS/SULT1E1 ratio correlates with prognosis; ie, patients with tumors negative for STS and positive for SULT1E1 had an improved outlook, whereas those positive for STS and negative for SULT1E1 were associated with unfavorable clinical outcome. Thus, estrogens generated through STS appear to contribute to CRC progression and poor survival. English et al (364, 365) also found STS activity to be increased in CRC tumors, and additionally 17βHSD-2 protein expression was frequently reduced with no alteration in aromatase activity; thus, increased E_1_ generated via STS, together with a fall in 17βHSD-2, should drive production of biologically active E_2_.

The evidence for DHEAS and DHEA in CRC incidence and proliferation is more obscure. Debate exists on whether there is any significant aromatase activity in the colon (358, 364) and, if it is present, whether it affects clinical outcomes (366). Therefore, local DHEAS desulfation would mostly be utilized for androgen production, and functional membrane ARs are present in colonic tumors (367). However, the effect of androgens in CRC is unclear. In vitro T induced apoptosis in CRC cell lines (315, 368), whereas DHEA enhances survival (315). In contrast, Tutton and Barkla (369) found that in vivo administration of T accelerated cell proliferation in the small intestine and induced colon cancer in rats, with CRC growth reduced after castration. This early study has been strongly supported recently by elegant studies demonstrating that T and DHT promote the development and proliferation of colon adenomas in rats and mice, whereas castration markedly protected colon adenoma formation (370).

In humans, studies exploring the effect of androgen treatment on the colon have been inconsistent. Alberg et al (371), examining serum from CRC patients, found that higher circulating DHEAS concentrations in men were slightly associated with a decreased risk of CRC. Supporting this finding, a large study of 107 859 prostate cancer patients explored CRC incidence and androgen deprivation therapy (372). Initial results showed orchiectomy caused the highest incidence of CRC, followed by GnRH agonist therapy and men with no androgen deprivation therapy. CRC risk increased with the length of time a patient was subjected to androgen deprivation therapy (373), and thus androgens may act like estrogens with both protective and cancer-promoting effects in the context of CRC.

### B. Aging

Serum DHEA and DHEAS decline with age, and at 70 years of age, circulating DHEAS concentrations have diminished by 90% compared with the peak levels achieved at ages 20–30 (374). Thus, there is widespread speculation about a causative role of DHEAS in age-related disease development and human longevity. In cross-sectional studies, low DHEA and DHEAS concentrations have been associated with geriatric syndromes, such as sarcopenia (375, 376), poor cognitive function (377), depression (378), cardiovascular disease (379), erectile dysfunction (380), and low sexual drive (381). Little is known about what triggers the gradual decline of DHEA and DHEAS, but because it accounts for 50% of androgens in men and 75% of estrogens in premenopausal women (382), delineating this effect is of significant importance in age-related research.

It is most likely that declining DHEA and DHEAS concentrations are associated with decreased adrenal production, rather than an alteration in DHEA metabolism (383). However, some evidence suggests that a relationship between DHEAS and DHEA is defined by activity of SULT2A1, the enzyme that converts DHEA to DHEAS (223), and that impairment of DHEA sulfation causes low DHEAS and concurrent androgen excess with high DHEA and androstenedione concentrations (237). Genetic variants of SULT2A1 do not appear to have an effect on individual DHEA and DHEAS concentrations or the DHEA/DHEAS ratio as a marker of DHEA sulfonation capacity (249). However, to date, no other research has been published on aged-induced alterations in SULT2A1 and STS activity, particularly in the adrenal gland; thus, conclusions on potential mechanisms behind the age-associated decline in DHEA and DHEAS are lacking.

## VII. Pharmacological Intervention

### A. STS inhibitors

Clearly, the ability to pharmacologically target STS has significant potential in a number of disease states. In cancer, where the desulfation of E_1_ and DHEA may play important roles in breast and prostate cancer, STS inhibitors may show significant promise (269). Furthermore, steroid dynamic studies reveal that DHEA and DHEAS can act as precursors for the formation of other steroids with estrogenic and androgenic properties, such as 5-androstenediol (Adiol). Evidence suggests that DHEAS (301) and Adiol (384) stimulate breast cancer cell proliferation in vitro, although other contradictory evidence suggests that DHEA may play a protective role against the disease (304,385). Interestingly, DHEAS concentrations in plasma are very high (Table 1); it is the most abundant steroid secreted by the adrenal cortex. Similar to E_1_S, it has a long plasma half-life (10–20 h), significantly longer than the unconjugated DHEA (386, 387). After hydrolysis via STS, DHEA undergoes further reduction to Adiol, an androgen steroid able to bind to the ER and cause mitogenesis (388). Therefore, due to the large plasma concentrations of the precursors of Adiol, this STS-affected pathway may play an important role in cancer tumorigenesis. Thus, inhibiting STS should not only block E_1_ synthesis, but also significantly limit androgen precursors. Indeed, in the first phase I clinical trial of an STS inhibitor, circulating androstenedione and T were significantly decreased in postmenopausal women with refractory hormone-dependent breast cancer (307). There have been several recent comprehensive and excellent reviews covering the development of STS inhibitors for various hormone-dependent malignancies (269, 389–391); thus, this section will only briefly examine and summarize the current status of STS inhibitor development.

The first STS inhibitor to demonstrate hepatic in vivo activity in a rat model was 667Coumate (STX64, Irosustat), a potent tricyclic coumarin-based sulfamate that irreversibly inhibits STS (392). This compound has shown excellent in vivo efficacy against E_2_S-driven breast cancer (393, 394) and endometrial cancer (357) and has shown promise in phase I clinical trials in female patients with hormone-dependent breast cancer (307). Currently, 667Coumate undergoes evaluation in hormone-dependent breast cancer patients in combination with aromatase inhibitors in phase I/II trials, and results should be published in late 2015.

The first-in-class success of 667Coumate has not dampened enthusiasm for further development of novel STS inhibitors. Recently, for example, several groups have developed derivatives of E_1_ sulfamate (395), 4-substituted E_1_ and E_2_ (396), and 17β-arylsulfonamides of 17β-aminoestra-1,3,5(10)-trien-3-ol (397). Others are investigating the potential for dual-acting compounds that target both STS and ERα (398) or STS and aromatase (399), and second-generation STS inhibitors have been shown to be effective against E_2_S-stimulated breast cancer in vivo (393). Recently, a compound, EM-1913, has shown efficacy against DHEAS desulfation and therefore inhibits effects in androgen-sensitive tissues (316).

### B. Modulation of sulfation

All human sulfotransferases need the atypical nucleotide PAPS as an active sulfate donor, and PAPS binding is highly conserved between distantly related members of this large gene family. Because PAPS has an adenosine moiety, kinase-directed and purine-based compound libraries have been used in the past to discover sulfotransferase inhibitors (400–402). To develop SULT isoform-specific inhibitors, bisubstrate analog-based approaches have been applied to various sulfotransferases (403, 404). Although these early studies have aimed at cytosolic sulfotransferases, more research activity may have been spent on heparan and chondrocyte sulfotransferases (80, 405–407).

The rate-limiting step for all sulfation reactions is provision of active sulfate in the form of PAPS, and the responsible PAPS synthases are recognized as fragile enzymes stabilized by the APS intermediate of PAPS biosynthesis (70, 72). APS interacts both with the sulfurylase and APS kinase domain and effectively suppresses PAPSS2 aggregation at low micromolar concentrations (72). Exploiting this principle of action for compound development may result in PAPS synthase-stabilizing compounds that may increase overall sulfation capacity.

## VIII. Future Directions

Historically, steroid sulfation was regarded as a mechanism to facilitate steroid circulatory transit and renal excretion. Research over the past few decades challenged this view because it became clear that circulating steroid sulfates (ie, DHEAS) are desulfated and thus act as a systemic reservoir for peripheral metabolism. This is especially important because peripheral or local steroidogenesis can thus occur in otherwise nonsteroidogenic tissues (ie, devoid of the P450 side chain-cleaving enzyme P450scc), such as the brain or in prostate cancer (408). Sulfation and desulfation represent a dynamic way of balancing the availability of free steroid hormones near target sites; however, these processes need to be tightly controlled in cells where steroid sulfotransferase and sulfatase are coexpressed to avoid a vicious cycle.

This review has made clear that steroid hormone action strongly relies on the intricate interplay of sulfation and desulfation processes as well as membrane transport of sulfated steroids. Studies simultaneously looking at all three of these processes are still lacking; there are no clear data on the factors that regulate these pathways, and subsequently their importance in many pathologies has most likely been overlooked. It is clear that the ratios between STS and SULTs will have profound consequences on local steroid metabolism, but research into how these ratios impact upon normal and diseased tissue remains to be done.

It would be of great interest to map the relative concentrations of sulfated and desulfated steroids in a tissue-specific manner under various physiological states. Whether MS imaging (409) may turn out to be useful in this regard depends on when it will reach spatial resolution on a single cell level. Furthermore, the accurate measurement of the intracellular fluctuations of both sulfated and nonsulfated steroids in both normal and pathological states would provide significant insights into STS, SULT, and OATP biology.

Furthermore, the direct biological effects of steroid sulfates are the subject of lively scientific debate. E_1_S may elicit biological effects in uterine endometrium that are not seen with E_2_ (15). As a neurosteroid, pregnenolone sulfate clearly exerts different effects than its nonsulfated counterpart, pregnenolone. Although unconjugated pregnenolone is a barbiturate-like agonist, pregnenolone sulfate can bind to and suppress the gamma-aminobutyric acid receptor acting as a picrotoxin-like antagonist (410). It is difficult to dissect the molecular roles of DHEA and its sulfate ester, DHEAS. Experimentally, it is challenging to discriminate between direct DHEAS effects and those caused by desulfation and downstream conversion to more potent androgens and estrogens. DHEAS has been reported to induce transcription of the abundant miR-21 in liver cell lines; however, this effect is clearly linked to both desulfation and conversion to more potent androgens and estrogens (411). Evidence accumulates that DHEAS may have physiological roles of its own—as a neurosteroid acting antagonistically to DHEA (408); it has a hormone-like activity on the spermatogenic GC-2 cell line by activating a Gα11-receptor (412) and has been shown to directly activate protein kinase Cβ in human neutrophils (413).

Pharmacological intervention on sulfation and desulfation pathways remains in its infancy. Although promising progress has been made with regard to STS inhibition, few pharmacological tools exist to selectively target individual SULTs or OATPs. The development of these inhibitors would not only be a boon for basic researchers but also would allow for the potential development of future drugs targeting sulfation/sulfate transportation, many of which are up-regulated in various pathologies.
